# Enhanced Localization and Orientation Estimations in Focal EEG Source Imaging Using SVD-Based Coordinate Transform

**DOI:** 10.1007/s10548-025-01154-7

**Published:** 2025-10-22

**Authors:** Joonas Lahtinen, Alexandra Koulouri

**Affiliations:** 1 Faculty of Information Technology and Communication Sciences, Tampere University, Korkeakoulunkatu 3, Tampere, 33014 Finland; 2 Institute for Mathematical Innovation, University of Bath, BA2 7AY Bath, Great Britain

**Keywords:** EEG, Source imaging, SVD, Sparsity constraints, Adaptive Lasso, Adaptive group Lasso

## Abstract

Accurate localization and orientation estimation of neural sources are crucial in electroencephalography (EEG) source imaging, particularly for focal brain activities. This study introduces an enhanced method that integrates a Singular Value Decomposition (SVD)-based coordinate transform to improve the performance of Hierarchical Adaptive *L*1-Regression (HAL1R). By applying the SVD transform to the lead field matrix columns corresponding to individual source locations, we derive physiologically meaningful orientation bases that align with the brain’s structural and functional properties. Enforcing sparsity into these bases mitigates orientation biases inherent in standard *L*1-norm algorithms applied in traditional Cartesian systems. Numerical simulations and somatosensory evoked potential (SEP) data validate the proposed approach, demonstrating improved localization stability and orientation accuracy compared to conventional methods, such as Adaptive Group LASSO, Unit Noise Gain (UNG) Beamformer, and Dipole Scanning (DS). The SVD-based HAL1R framework establishes a robust and generalizable methodology for EEG source imaging, enhancing its accuracy and utility in clinical and research settings, including pre-surgical planning and non-invasive cortical mapping.

## Introduction

Neuroelectric inverse problems arise in clinical brain imaging, where we aim to estimate the underlying activity from electroencephalography (EEG) measurement data (Niedermeyer and da Silva 2004). The measurements are most often taken non-invasively from the human scalp. In a scenario like this, there are always more possible electromagnetic states inside the brain than one has measurement sensors, which makes the problem mathematically ill-posed and solutions non-unique (Hämäläinen et al. 1993). To counteract the ill-posedness, we can make the inverse problem well-posed by adding *a priori* information. This information can be given in the form of distributions assigned for the brain activity. A modeling approach of this kind is called *Bayesian modeling* (Kaipio and Somersalo 2004).

The inversion algorithm designed to solve the neuroelectromagnetic inverse problem can be roughly divided into two classes: filtering methods and distributional methods. Filtering methods scan through every source position and try to fit dipolar activity to each location separately. Distributional methods, on the other hand, solve a field of dipolar sources that best suits the given object function or point estimate of the posterior distribution in the Bayesian modeling framework. While distributional methods are good for estimating source extends and more widespread sources, they perform rather poorly in orientation estimation compared to filtering methods (Lin et al. 2006; Lahtinen et al. 2022; Buschermähle et al. 2024). The main reason for this lies in the modality-based biases as the source’s orientation affects the signal detection (Dassios et al. 2007). This means that some source directions yield a stronger data signal. If the inversion method is not orientationally balanced, the estimations will favor directions corresponding to the strongest signals.

The orientation estimation, along with positional, is an important aspect in designing optimal tES stimulation treatment, as we need to know beforehand the orientation of the targeted neuronal population (Khan et al. 2022, 2023; Becker et al. 2015). Moreover, incorporating an orientation estimation technique or constraint has been shown to increase the estimation accuracy (Lin et al. 2006; Bonaiuto et al. 2020). For example, suppose we want to estimate a source appearing on a wall of a sulcus. In that case, the correctness of the positional estimation can depend on the accuracy of orientational estimation, as the wrongly oriented source could be estimated to be at the opposing wall. Knowledge of the correct sulcal wall, i.e., correct orientation, could be important in identifying the epileptogenic side (Salayev et al. 2006; Güllmar et al. 2010).

In this paper, we propose a singular value decomposition-based idea to transform the standard coordinate system from the MRI data into a physiological and structural-based system that allows more accurate recovery of the orientation of the source activity when we employ sparsity priors that heavily depend on the selected coordinate system.

Here, we apply the condition to the focal estimator called the Hierarchical Adaptive *L*1-Regression (HAL1R) algorithm and compare the destination accuracy to the Adaptive Group LASSO version of it (GLASSO), which can be seen as an orientational non-biased version of HAL1R since its prior does not depend on the orientation of the source. These new methods are further compared to the Unit Noise Gain (UNG) Beamformer and Dipole Scanning (DS), both of which are shown to provide good location and orientational estimates (Buschermähle et al. 2024). The methods are compared using numerical simulations and real data of somatosensory evoked potentials, aiming to recover the dipolar cortical P20/N20 activity. The somatosensory evoked potentials were chosen because they are widely studied, and our knowledge of their originators is relatively high compared to other evoked potentials. Also, the localization of the primary somatosensory cortex is a task that has a freestanding clinical value regarding surgery close to the said area or general non-invasive study of the human cortical function (Nitsche et al. 2003).

The numerical result shows that the singular value decomposition-based modification of HAL1R improves its localization and orientation estimation accuracy compared to the non-modified one. Also, in the case of a tangential source, SVD-based HAL1R and Adaptive Group LASSO meet the accuracy of Unit Noise Gain Beamformer and Dipole Scanning. The estimations from the real subject’s EEG data show that Adaptive Group LASSO match accurately to the description of the cortical 20 ms activity given in Hari et al. (1993), Buchner (1995). The same location and slightly different orientation are provided by SVD-based HAL1R, while the Basic HAL1R estimate is off by orientation, and filtering methods localize and orient the source against the literature.

## Inversion Methods

In the neuroelectric inverse problem (Kaipio and Somersalo 2004), the observation model is

1 $$\begin{aligned} {\textbf {y}}=L{\textbf {x}}+\tilde{{\textbf {n}}}, \end{aligned}$$

where ${\textbf {y}}\in \mathbb {R}^m$ is the measurement vector, $\tilde{{\textbf {n}}}\in \mathbb {R}^m$ is the additive Gaussian measurement noise ($\tilde{{\textbf {n}}}\sim \mathcal {N}({\textbf {0}},C)$), ${\textbf {x}} \in \mathbb {R}^{3n}$ describes the neural activity modeled as *n* dipoles distributed in the brain and and $L\in \mathbb {R}^{m\times 3n}$ is the lead field matrix (Weinstein et al. 2000). Here a dipole source at location *k* is described by a vector ${\textbf {x}}_k=(x_{3(k-1)+1},x_{3(k-1)+2},x_{3(k-1)+3})$.

### Unit Noise Gain (UNG) Beamformer

UNG beamformer, also known as Borgiotti-Kaplan beamformer, is a beamforming strategy aiming at unbiased localization and orientation by its constraints (Westner et al. 2022; Buschermähle et al. 2024). The UNG beamformer is obtained as a filter solution by solving the following minimization problem at *k*th source location:

2 $$\begin{aligned} \begin{aligned} \min _{{\textbf {w}}_k\in \mathbb {R}^m}&{\textbf {w}}_k^T\Sigma {\textbf {w}}_k\\ \text {subject to }&{\textbf {w}}_k^TL_k\varvec{\theta }=\tau \quad \text {and}\quad {\textbf {w}}_k^T{\textbf {w}}_k=1, \end{aligned} \end{aligned}$$

where $\Sigma \in \mathbb {R}^{m\times m}$ is the error covariance matrix in measurement space, $L_k\in \mathbb {R}^{m\times 3}$ is a sub-matrix of the lead field corresponding to *k*th source location, $\varvec{\theta }\in \mathbb {R}^3$ is the source orientation vector and $\tau$ is a scalar constant with a value specified by the second constraint. The filtering problem has the solution of the form

3 $$\begin{aligned} {\textbf {w}}_k(\varvec{\theta })=\frac{\Sigma ^{-1}L_k\varvec{\theta }}{\sqrt{\varvec{\theta }^TL_k^T\Sigma ^{-2}L_k\varvec{\theta }}} \end{aligned}$$

that is derived in Appendix A. The optimal orientation is assumed to maximize the signal power, which yields the orientation solution:

4 $$\begin{aligned} \hat{\varvec{\theta }}= \varvec{\vartheta }_{\text {max}}\left\{ \left( L_{k}^{T}\Sigma ^{-2}L_{k}\right) ^{-1}L_{k}^{T}\Sigma ^{-1}L_{k}\right\} , \end{aligned}$$

where $\varvec{\vartheta }_{\text {max}}$ denotes the eigenvector corresponding to the maximum eigenvalue of the expression inside the curly brackets (Sekihara and Nagarajan 2008).

### Dipole Scan

The Dipole scan (DS) is based on the best-fit solution that minimizes the residual variance between the measured data and a dipole forward mapped to measurement space (Fuchs et al. 1998). The goodness of the fit for the *k*th source location is defined as

5 $$\begin{aligned} g_k=1-\frac{\left\| {\textbf {y}}-L_kL_k^+{\textbf {y}}\right\| ^2_2}{\left\| {\textbf {y}}\right\| _2^2}, \end{aligned}$$

where $(\cdot )^+$ is the pseudoinverse operator and **y** denotes the *m*-dimensional measurement vector.

### Hierarchical Adaptive *L*1-Regression

In the hierarchical Bayesian method called HAL1R, we employ the multivariable Laplace prior

6 $$\begin{aligned} \text {Lap}({\textbf {x}}\mid \varvec{\gamma },{\textbf {0}})=\prod _{i=1}^{3n}\frac{\gamma _i}{2}\exp \left( -\gamma _i\left| x_i\right| \right) , \end{aligned}$$

where $\gamma _i$ denotes a component of hyperparameter vector $\varvec{\gamma }\in \mathbb {R}^{3n}$. This prior distribution allows the sources to be distributed in a small region reflecting a focal brain activity (Lahtinen et al. 2022). To allow greater flexibility to the prior model instead of fixed values, hyperparameters $\gamma _i$ are random variables that follow the gamma distribution:

7 $$\begin{aligned} \text {Ga}(\varvec{\gamma }\mid \varvec{\beta },\varvec{\theta })=\prod _{i=1}^{3n}\frac{\gamma _i^{\beta _i-1}\exp (-\gamma _i/\theta _i)\theta _i^{-\beta _i}}{\Gamma (\beta _i)}, \end{aligned}$$

where $\Gamma (\cdot )$ is the gamma function and $\beta _i$ is the shape and $\theta _i$ is shape parameters respectively. For Bayesian inference, the posterior distribution is

8 $$\begin{aligned} \begin{aligned} p ({\textbf {x}},\varvec{\gamma }\mid {\textbf {y}})&\propto p ({\textbf {y}}\mid {\textbf {x}})p ({\textbf {x}}\mid \varvec{\gamma })p (\varvec{\gamma })\\&=\mathcal {N}({\textbf {y}}\mid L{\textbf {x}},C)\times \text {Lap}({\textbf {x}}\mid \varvec{\gamma },{\textbf {0}})\times \text {Ga}(\varvec{\gamma }\mid \varvec{\beta },\varvec{\theta }). \end{aligned} \end{aligned}$$

where $p({\textbf {y}}\mid {\textbf {x}})=\mathcal {N}({\textbf {y}}\mid L{\textbf {x}},C)$ is a Gaussian likelihood considering measurement noise Gaussian with zero mean and covariance $C\in \mathbb {R}^{m\times m}$.

In practice, we can compute point estimates like Maximum a posteriori (MAP) by using, for example, *expectation maximization* (EM) and approximating via Majorization-Minimization using Local Quadratic Approximation (MM-LQA) (Kim et al. 2018). In the current work, by applying EM, we solve recursively the optimization problem

9 $$\begin{aligned} \hat{{\textbf {x}}}^{(t+1)}=\underset{{\textbf {x}}\in \mathbb {R}^{3n}}{\text {arg max }}\, \mathbb {E}_{p (\varvec{\gamma }\mid {\textbf {x}}^{(t)})}[\log p \left( {\textbf {x}},\varvec{\gamma }\mid {\textbf {y}}\right) ], \end{aligned}$$

where $t\ge 1$ indicates an arbitrary step. The expectation is

10 $$\begin{aligned} \mathbb {E}_{\log p (\varvec{\gamma }\mid \hat{{\textbf {x}}}^{(t)})}[ p \left( {\textbf {x}},\varvec{\gamma }\mid {\textbf {y}}\right) ]=\int _\gamma \log p \left( {\textbf {x}},\varvec{\gamma }\mid {\textbf {y}}\right) p (\varvec{\gamma }\mid \hat{{\textbf {x}}}^{(t)}) \;\text {d}\gamma . \end{aligned}$$

This results in

11 $$\begin{aligned} {\hat{\gamma }}_i^{(t)}&=\mathbb {E}_{p (\gamma _i\mid \hat{{\textbf {x}}}^{(t)})}\left[ \gamma _i\right] , \end{aligned}$$

12 $$\begin{aligned} \hat{{\textbf {x}}}^{(t+1)}&=\underset{{\textbf {x}}\in \mathbb {R}^{3n}}{\text {arg max}}\left\{ \log p \left( {\textbf {y}}\mid {\textbf {x}}\right) +\mathbb {E}_{p (\varvec{\gamma }\mid \hat{{\textbf {x}}}^{(t)})}\left[ \log p ({\textbf {x}}\mid \varvec{\gamma })\right] \right\} , \end{aligned}$$

which for the posterior (8) becomes

13 $$\begin{aligned} {\hat{\gamma }}_i^{(t)}&=\frac{\beta _i+1}{\left| {\hat{x}}_i^{(t)}\right| +\theta _i},\quad \forall \, i=1,\cdots , 3n, \end{aligned}$$

14 $$\begin{aligned} \hat{{\textbf {x}}}^{(t+1)}&=\underset{{\textbf {x}}\in \mathbb {R}^{3n}}{\text {arg min}}\left\{ \frac{1}{2}({\textbf {y}}-L{\textbf {x}})^TC^{-1}({\textbf {y}}-L{\textbf {x}})+\sum _{i=1}^{3n}{\hat{\gamma }}_i^{(t)}\left| x_i\right| \right\} . \end{aligned}$$

Here, an instance of the previous L1 norm optimization problem is solved using the EM-MM-LQA algorithm due to the large variable dimension. This hierarchical modeling has been shown to accurately estimate focal epilepsy sources from interictal data of epilepsy patients suffering from focal cortical dysplasia (Lahtinen et al. 2023). We note that HAL1R is closely related to the method proposed in Calvetti et al. (2009). However, the distinction is that the model uses a gamma prior for the hyperparameter $\varvec{\gamma }$ of the Laplacian prior instead of a Gaussian prior.

### Hierarchical Adaptive Group Regression

As an attempt to make an orientation-unbiased solver, one could replace the heavily orientation-dependent *L*1-norm with the *L*2-norm of the dipole components (orientation invariant option) (Koulouri 2015; Koulouri et al. 2016). In that case, the prior (6) is re-written as

15 $$\begin{aligned} \text {Lap}({\textbf {x}}\mid \varvec{\gamma },{\textbf {0}})=\prod _{k=1}^{n}\left( \frac{\gamma _k}{2}\right) ^3\exp \left( -\gamma _k\left\| {\textbf {x}}_k\right\| _2\right) , \end{aligned}$$

where $\Vert {\textbf {x}}_k\Vert _2=\sqrt{x_{3(k-1)+1}^2+x_{3(k-1)+2}^2+x_{3(k-1)+3}^2}$ (Koulouri et al. 2017). Following similar procedures as in the previous section, we get the following optimization problem to be solved recursively:

16 $$\begin{aligned} {\hat{\gamma }}_k^{(t)}&=\frac{\beta _i+3}{\left\| \hat{{\textbf {x}}}_k^{(t)}\right\| _2+\theta _i},\quad \forall \, k=1,\cdots , n, \end{aligned}$$

17 $$\begin{aligned} \hat{{\textbf {x}}}^{(t+1)}&=\underset{{\textbf {x}}\in \mathbb {R}^{3n}}{\text {arg min}}\left\{ \frac{1}{2}({\textbf {y}}-L{\textbf {x}})^TC^{-1}({\textbf {y}}-L{\textbf {x}})+\sum _{k=1}^{n}{{\hat{\gamma }}_k^{(k)}}\left\| {\textbf {x}}_k\right\| _2\right\} . \end{aligned}$$

Here we set ${\textbf {x}}^{(0)}={\textbf {0}}$. One estimate of the group optimization problem can be solved iteratively using again EM or interior point methods, for example.

## Singular Value-Based Orientation System

It is well known that applying the *L*1-norm to the source components in the Cartesian coordinate system (MRI-based system) favors source reconstructions in one of these directions. This can be concluded when considering the bias-variance trade-off of the LASSO regression (Li et al. 2012). As MRI-based directions are the same for each source location, they have little to no relevance in the physiological directionality of the neural activity.

**Fig. 1:**
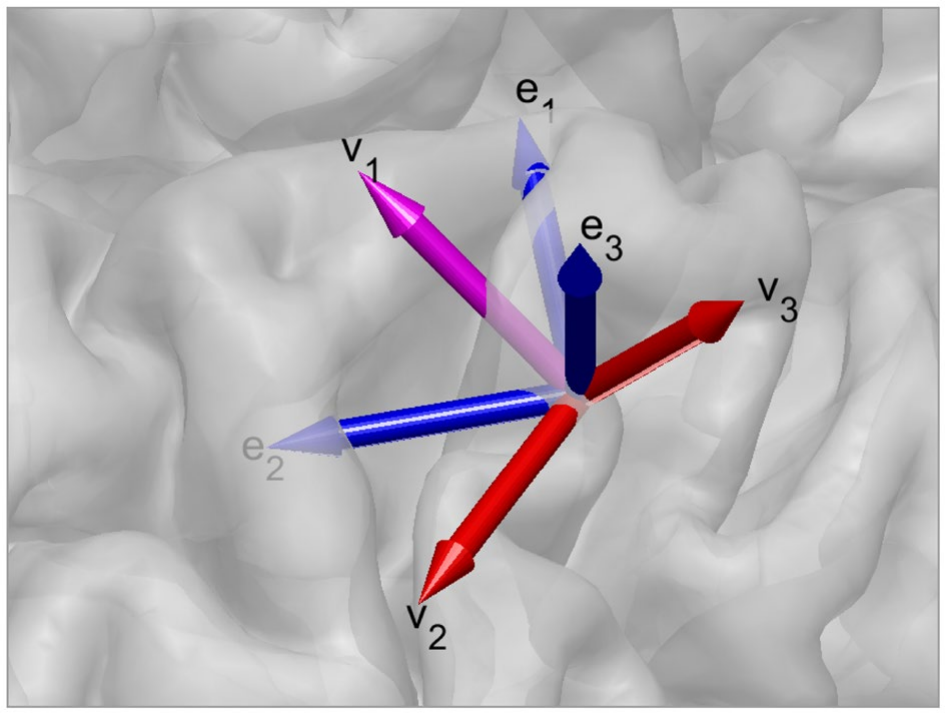
With a lead field system direction by cartesian coordinates $({\textbf {e}}_1,\, {\textbf {e}}_2,\, {\textbf {e}}_3)$, represented as blue arrows, and by applying SVD to the sub-lead field $L_k\in \mathbb {R}^{m\times 3}$, we can obtain basis $({\textbf {v}}_1,\, {\textbf {v}}_2,\, {\textbf {v}}_3)$, shown by magenta and red arrows, that we use the basis for the sparsity constraint instead. Due to the nature of the SVD, its basis is always directed by its principal directions of the sub-leadfield’s $L_k$ output strength in the signal space. For example, the figure shows that the strongest system output of $L_k$ is obtained in a tangential direction (magenta arrow) and the weakest direction is radial

**Fig. 2:**
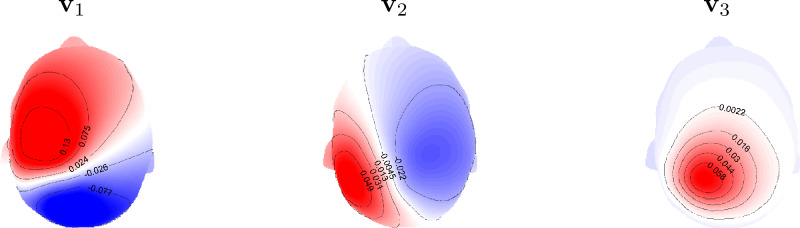
Topography plots of the simulated data produced by the three physiologically relevant basis vectors obtained by SVD of the location-specific lead field at the postcentral gyrus. The topographies provide us with information about the three orientations i.e. $\text {v}_1$ and $\text {v}_2$ are tangential and $\text {v}_3$ is radial (normal) with respect to the head surface

The alternative is to apply anatomical information to penalize the directions in the *L*1-norm (Lin et al. 2006). However, the main difficulty arising in this approach is that precise knowledge of the subject’s cortical and brain structure is essential and needs to be analyzed throughout. In this work, we investigate and apply an easy approach to incorporate implicit anatomical information encoded in the lead field to the *L*1-norm by employing the right-hand side principle eigenvectors of the singular value decomposition (SVD) of the lead field columns corresponding to each source location. As shown in Fig. 1, the right-hand eigenvectors provide a way to easily rotate the MRI coordination system to an anatomically based coordination system. The topographies of simulated data caused by a single dipole on the somatosensory cortex at each of the principal directions are demonstrated in Fig. 2. Therefore, here we apply *L*1-sparsity in the new rotated directions.

In particular, by applying SVD in every sub-lead field $L_k=U_kS_kV_k^T\in \mathbb {R}^{m\times 3}$ corresponding to a location $k=1,\ldots ,n$ and $V_k=({\textbf {v}}_1^{(k)},{\textbf {v}}_2^{(k)},{\textbf {v}}_3^{(k)}$), we define a set of basis functions i.e. $\grave{V}=\left[ \grave{{\textbf {v}}}_1,\,\cdots \, ,\grave{{\textbf {v}}}_{3n}\right] \in \mathbb {R}^{3n\times 3n}$ such that $\grave{{\textbf {v}}}_{3(k-1)+i}:={\textbf {e}}_k\otimes {\textbf {v}}_i^{(k)}$ with ${\textbf {e}}_k\in \mathbb {R}^n$ being a unit vector with 1 at index *k*, *i* is the index of the cartesian direction in the original forward model, and $\otimes$ is the Kronecker product.

Therefore, we can set ${\textbf {x}}=\grave{V}\varvec{\xi }$ and apply HAL1R for the new variable $\varvec{\xi }$, i.e.

18 $$\begin{aligned} {\hat{\gamma }}_i^{(t)}&=\frac{\beta _i+1}{\left| {\hat{\xi }}_i^{(t)}\right| +\theta _i},\,\forall \,i=1,\ldots ,3n \end{aligned}$$

19 $$\begin{aligned} \hat{\varvec{\xi }}^{(t+1)}&=\underset{\varvec{\xi }\in \mathbb {R}^{3n}}{\text {arg min}}\left\{ \frac{1}{2}({\textbf {y}}-A\varvec{\xi })^TC^{-1}({\textbf {y}}-A\varvec{\xi })+\sum _{i=1}^{3n}{\hat{\gamma }}_i^{(t)}\left| \xi _i\right| \right\} \end{aligned}$$

where $$A=L\grave{V}$$.

### Hyperparameter Selection of the Hierarchical Bayesian Methods

Due to the usage of the gamma hyperprior model, we need to decide the shape parameters $\beta _i$ and scale parameters $\theta _i$ for Basic HAL1R, Adaptive Group LASSO, and SVD-based HAL1R. To ensure a fair comparison, we use the same optimality condition-based approach to select these parameters. The optimality condition gives us a way to ensure that the initial guess is not everywhere zero in a LASSO-based algorithm.

Since these models do not have a gradient at zero, we write the optimality condition in the subgradient sense as:

20 $$\begin{aligned} {\textbf {0}}\in \left\{ \partial \left( \log p \left( {\textbf {y}}\mid {\textbf {x}}\right) +\mathbb {E}_{p (\varvec{\gamma }\mid \hat{{\textbf {x}}}^{(t)})}\left[ \log p ({\textbf {x}}\mid \varvec{\gamma })\right] \right) \right\} . \end{aligned}$$

This notation means that the zero vector should belong to the set formed by the subgradient $\partial (\cdot )$ of the objective function.

Considering first the Basic HAL1R, the subgradient of its objective function is

21 $$\begin{aligned} {\textbf {0}}\in \left\{ -L^TC^{-1}({\textbf {y}}-L{\textbf {x}})+{\textbf {p}}\right\} , \end{aligned}$$

where ${\textbf {p}}$ is the subgradient of the penalty term:

22 $$\begin{aligned} p_i={\left\{ \begin{array}{ll} \lbrace {\hat{\gamma }}_i\rbrace ,& x_i>0,\\ \lbrace -{\hat{\gamma }}_i\rbrace ,& x_i<0,\\ \left[ -{\hat{\gamma }}_i,\, {\hat{\gamma }}_i\right] ,& x_i=0, \end{array}\right. } \end{aligned}$$

where ${\hat{\gamma }}_i$ are the hyperparameter values at the optimum point. From this, we can deduce that if

23 $$\begin{aligned} \left| \left[ L^TC^{-1} {\textbf {y}}\right] _i\right| \le {\hat{\gamma }}_i, \end{aligned}$$

then $x_i=0$ for all $i=1,\cdots 3n$.

Similarly, the subgradient form of the optimality condition in the case of Adaptive Group LASSO reads

24 $$\begin{aligned} {\textbf {0}}\in \lbrace -L_i^TC^{-1}({\textbf {y}}-L_i{\textbf {x}}_i)+{\hat{\gamma }}_i\mathbb {B}_3({\textbf {0}};1)\rbrace , \end{aligned}$$

where $\mathbb {B}_3({\textbf {0}};1)$ denotes a closed 3-dimensional unit ball at the origin, i.e.:

25 $$\begin{aligned} \mathbb {B}_3({\textbf {0}};1)=\left\{ {\textbf {p}}\in \mathbb {R}^3:\left\| {\textbf {p}}\right\| _2\le 1 \right\} . \end{aligned}$$

Now, the optimality condition has the form of

26 $$\begin{aligned} \left\| L_i^TC^{-1} {\textbf {y}}\right\| _2\le {\hat{\gamma }}_i, \end{aligned}$$

for $$i=1,\cdots , n$$ and $$L_i\in \mathbb {R}^{m\times 3}$$.

By denoting our modified lead field *A* in terms of its sub-SVD at a single source location and using the exact procedure as in the case of Basic HAL1R, we get

27 $$\begin{aligned} \left| \sigma _i{\textbf {y}}^T{\textbf {u}}_i\right| \le {\hat{\gamma }}_i, \end{aligned}$$

where $\sigma _i$ is the *i*th singular value and ${\textbf {u}}_i$ is the corresponding left singular vector.

As we have two parameters, one equation alone is not enough to recover both. So, in addition, we use an assumption of 10 nAm source strength that corresponds roughly to the total activity of tens of thousands of simultaneously firing neurons in one region (Hämäläinen et al. 1993). Mathematically, for example, in the case of HAL1R, we require that $\theta _i/\beta _i$ corresponds to the expected activity strength; also, within this reasonable activity scale, the optimality conditions for zero solutions will not hold.

## Subject and EEG Data

The dataset used in the experiment was obtained from a 49-year-old right-handed male with no history of psychiatric or neurological disorders. MRI data was used to construct a realistic model of his head. The dataset contains a defaced head model and montage-averaged EEG recordings. The MRI dataset, from which the head model was constructed, was measured by MAGNETOM Prisma scanner 3.0 T (Release D13, Siemens Medical Solutions, Erlangen, Germany) with T1 and T2-weighting (T1W/T2W) fast gradient-echo pulse sequence. SEP measurements were performed using 80 AgCl sintered ring electrodes (EASYCAP GmbH, Herrsching, Germany) with 74 EEG channels in the standard 10–10 system. A notch filter was applied to remove the interference caused by harmonics of the 50 Hz power line frequency and the 60 Hz monitor from which the subject watched a video during the measurement to reduce otherwise prominent alpha-activity. A sampling rate of 1200 Hz and an online low-pass filter at 300 Hz were used. 1200 stimuli were recorded for averaging, following the guidelines for spinal and subcortical SEPs (Cruccu 2008). The dataset is openly available (Piastra et al. 2020).

## Experiments

**Fig. 3:**
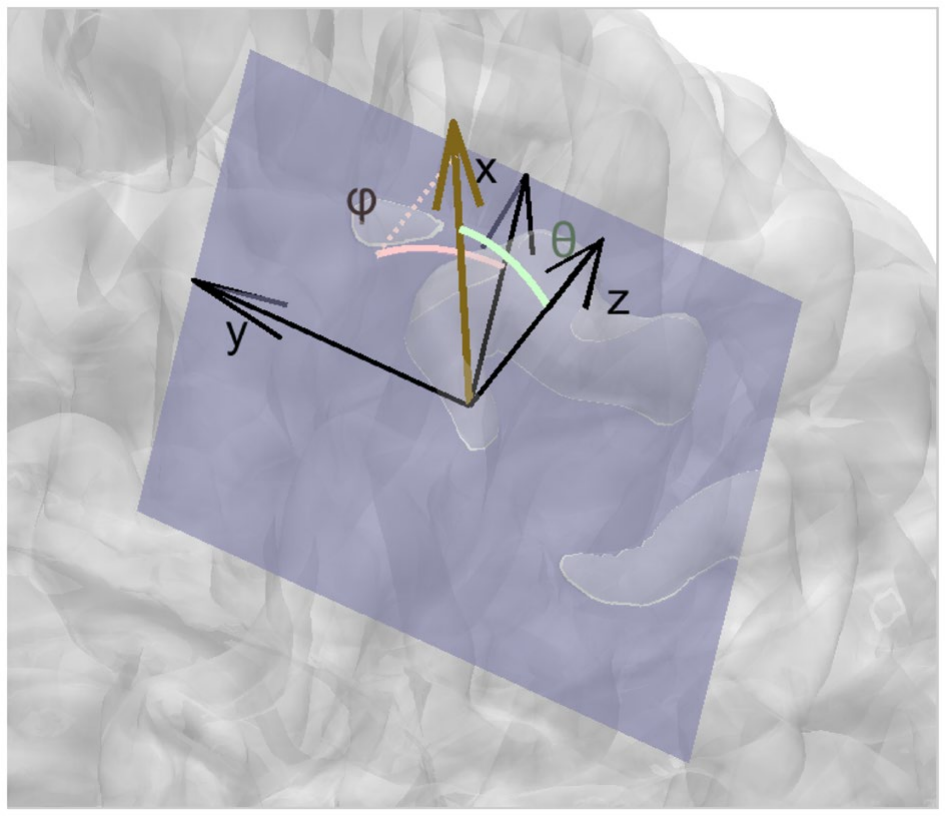
The used reference system for directions and angles. The dark yellow arrow represents a dipole moment, and its azimuthal angle $\varphi$ and polar angle $\theta$ are computed with respect to the blue-gray tangential plane of that source location

**Fig. 4:**
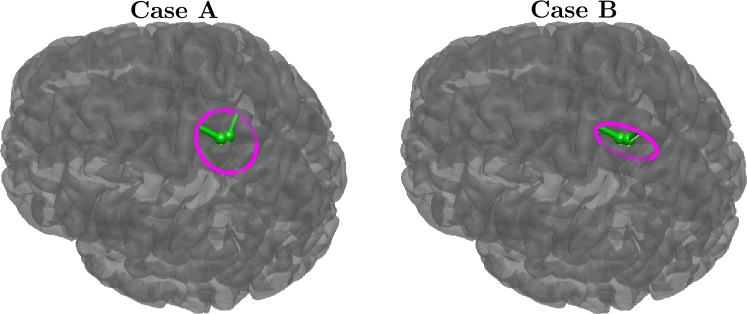
The orientational principal vectors (green arrows) and the orientation contour (magenta circle) for simulated Case A with both directions being tangential and Case B with the radial contribution. The rotation starts from the anterior direction and then goes towards the medial direction in Case A, and it starts from the anterior direction and goes to the radial direction in Case B

We use numerical simulations in addition to real somatosensory P20/N20 peak data to cover its cortical originator’s location and orientation. In the first numerical simulations, a head model with 80,000 source positions and 0.8 mm mesh accuracy was used to create simulated data from a dipolar activity at Broadmann area 3b, where the dipole orientation was varied across the tangent plane of the scalp normal (Case A) and its normal plane on anterior and normal directions (Case B), the reference system is illustrated in Fig. 3. In both cases, we let the orientation trip around the full 360 degrees, starting from the anterior direction. The orientation error is measured with the azimuthal angular error $\Delta \varphi$ and polar angular error $\Delta \theta$ of the local coordinates at the position of the true source that is illustrated in Fig. 4. The following process can be used for measuring angular errors: first, the angular error is measured on the tangent plane, then the dipole estimate is rotated to the correct xy-plane angle, and after this, the absolute value of the polar angle difference can be calculated. Finally, we plotted graphs of each inversion method’s localization error in millimeters and orientational errors in degrees, plotted using the function of the true dipole’s orientation. The compared methods are introduced in Sect. "Inversion Methods".

In the second numerical simulation, we gathered statistical dipole estimation error data of all compared methods using the same error measures as in the first numerical simulation. A total of 2,000 different source cases (orientation and position) were used to create synthetic data samples for (A) tangentially oriented and (B) radially oriented sources. The experiment was repeated with 5 and 15 % of noise. The results are presented using histograms. In numerical simulations only, to demonstrate the necessity to embed the SVD into the Bayesian model rather than use the two-step approach to estimate the location first and then the orientation known from fitting approaches, we provide results with "Fitted-GLASSO" named method that uses orientation-neutral Group LASSO to estimate the location and then SVD to provide the orientation that match best with the data.

**Fig. 5:**
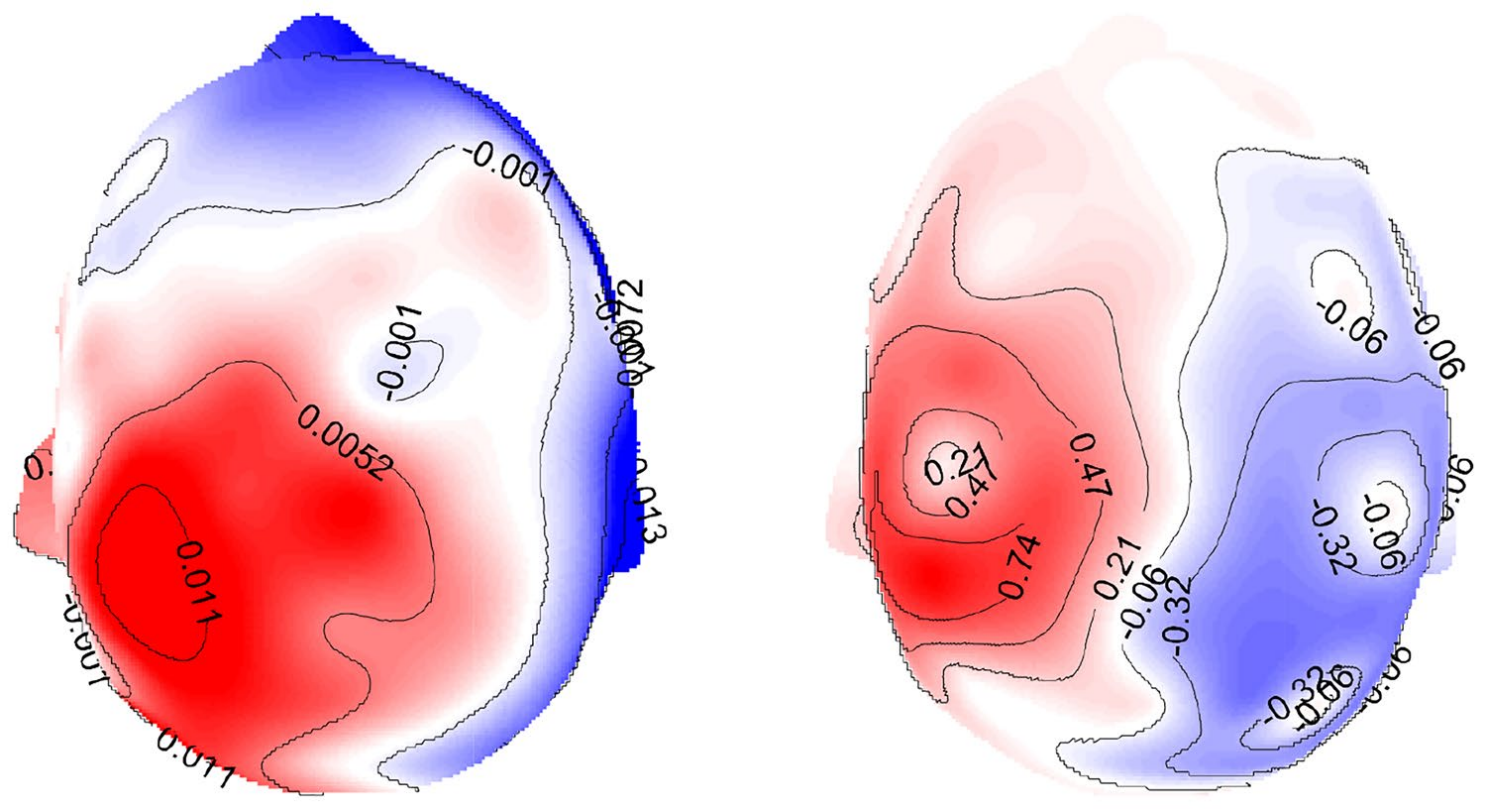
Topography plotted from the EEG measurements from the subject (left) and an example of simulated data (right)

With real data, we aim to recover the cortical P20/N20 originator that lies on the posterior wall of the central sulcus and should have a tangential orientation (Fuchs et al. 1998). As we do not have strong orientational ground truth, we assess the results reflecting the results from numerical simulations. As our results, we present the estimation distribution on the neocortex to asses the localization and estimated extent of the source, and the orientation estimations are presented as cone plots at the region of the maximum estimated source magnitude. An example case of the real topography and topography of noisy simulation is presented in Fig. 5.

## Results

### Simulations

**Fig. 6:**
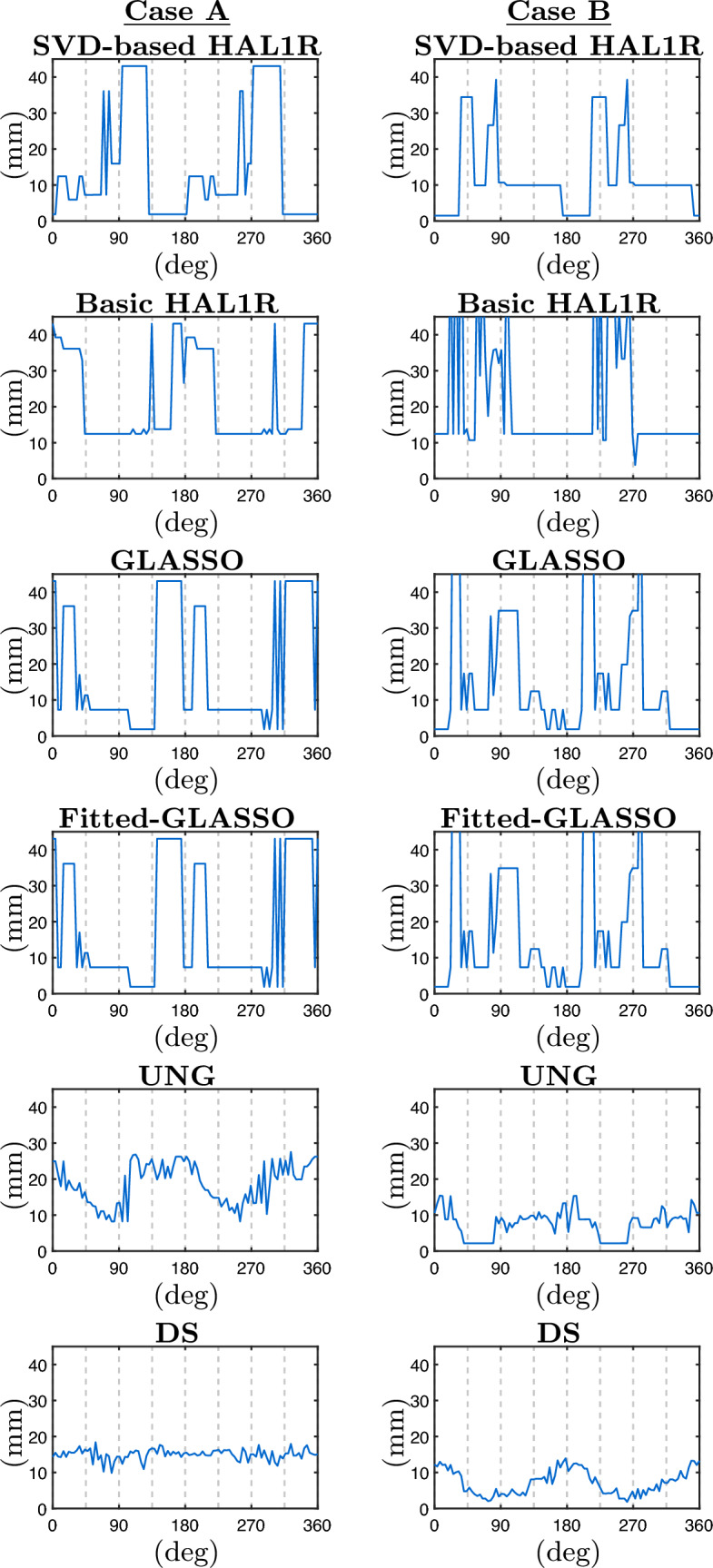
Localization error in millimeters (blue graphs) plotted as a function of the rotated degrees. The location of the true source and the orientation planes are illustrated in Fig. 4. The left column presents the results for Case A, and the right column presents the results for Case B. Different rows illustrate the results of different methods

**Fig. 7:**
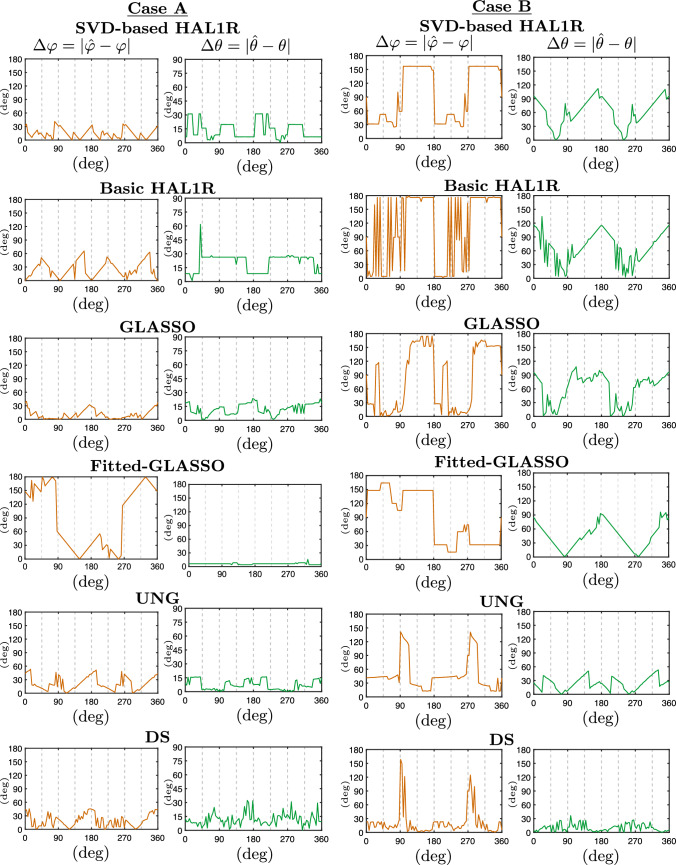
Orientation errors in degrees for the azimuthal angle $\varphi$ (orange curves) and polar angle $\theta$ (green curves) plotted as a function of the rotated degrees along the principal axes illustrated in Fig. 4. The left column presents the results for Case A, and the right column presents the results for Case B. Different rows illustrate the results of different methods

The simulations were divided into two cases: Case A, where the source is tangentially oriented, and Case B, where the source has radial contribution, such that in 90 and 270 degrees, the source is fully radial, and 0, 180, and 360 are fully tangential with respect to the scalp. The localization estimations are represented by blue curves in Fig. 6. The orientation error measured by an azimuthal and polar angle is presented in Fig. 7, where the azimuthal error is shown in orange graphs and the polar error in green graphs. The main columns labeled "Case A" and "Case B" present the results for the named true source setup. The main columns are divided into two sub-columns. The left sub-columns of the orientation error figure show the azimuthal errors, and the right sub-columns display the polar angle errors.

The estimations show satisfying localization with the proposed SVD-based HAL1R and GLASSO, with some orientation intervals in Case A. The UNG beamformer and Dipole Scan showed satisfying accuracy throughout the experiment. The original HAL1R and Adaptive Group LASSO localization is satisfying at best, but their behavior is highly unstable. The most stable localization error is obtained with SVD-based HAL1R and DS. At the same time, SVD-based HAL1R provides almost perfect localization when the dipole is oriented chiefly sideways with respect to the virtual subject’s front. Interestingly, opposite orientation-dependent behavior can be obtained with Basic HAL1R, Group LASSO, and UNG. Orientation estimations in Fig. 7 display surprisingly similar azimuthal estimation behavior. In Case A, the azimuthal error stays under 60 degrees with all the methods except the basic HAL1R, and seems to peak at exactly x and y direction sources. The polar angular errors, on the other hand, are different for each method. The most moderate errors are obtained with SVD-based HAL1R, Group LASSO, and UNG, from which UNG provides the most accurate estimations under 15-degree errors. The polar angle error of SVD-based HAL1R is otherwise low but peaks slightly when the source is directed along the MRI-based axes.

In Case B (right column in Fig. 6), stable localization estimations are obtained with SVD-based HAL1R and Basic HAL1R. SVD-based HAL1R, UNG, and DS exhibited good localization estimation accuracy. UNG and DS prefer radially oriented sources, but the same cannot be said about the compared hierarchical Bayesian methods. While the localization estimations are better than in Case A, the orientation estimations are worse. The error peaks at 90 and 180 degrees in azimuthal angle are due to the error reaching the indefiniteness point, where the error estimation gets more sensitive. Most accurate azimuthal estimations are obtained with DS and Group LASSO, which are, disregarding the spikes, under 30 degrees. UNG provided a relatively stable 45-degree error, and Basic HAL1R is highly unstable. The best polar angle estimations are obtained with DS. The polar angle estimations of the hierarchical Bayesian methods are strikingly similar.

**Fig. 8:**
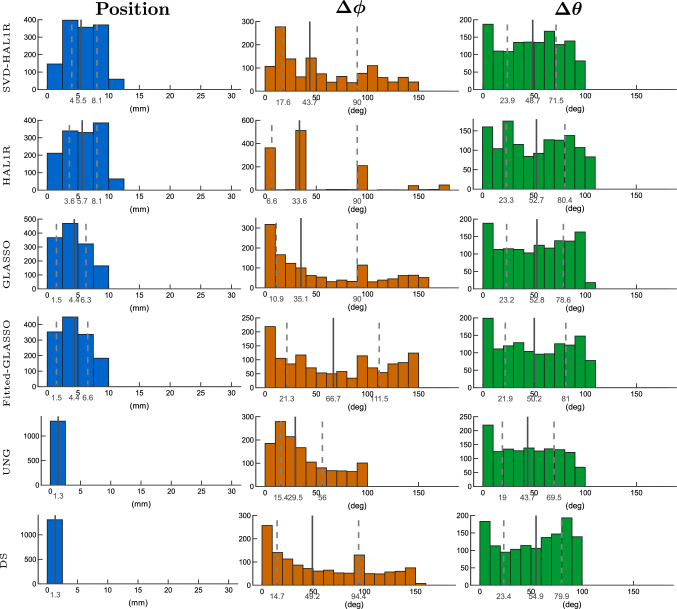
Histogram presentation of dipole estimation errors of 2,000 radially oriented cortical sources under 5 % of measurement noise. The mean, first, and third quartiles are presented on the histograms: the mean by a solid line and the other two quartiles by dashed lines. Blue histograms present the dipole localization errors, azimuthal angle errors are displayed by orange histograms, and green histograms provide the polar angle error distribution. Each row is dedicated to an individual source estimation method introduced in Sect. "Inversion Methods". Fitted-GLASSO is the demonstrative method that uses orientationally unbiased Group LASSO to estimate the location after which the orientation is sought out by using SVD

Results gathered from 2,000 different radial source scenarios with 5 % of noise in Fig. 8 show a less than 1 cm median source localization error within inter quartile range presented with dashed vertical lines around the mean presented by solid grey line on the histograms presented on the left and colored by blue. The most accurate localization among all is obtained with UNG and DS. In this case, UNG has the lowest orientational error among the methods (mean azimuthal error of 29.5 deg and polar angle error of 43.7 deg), with its distribution mass concentrated towards zero error, as shown in the orange azimuthal angle error histograms in the middle and green polar angle error histograms on the right. However, DS exhibits one of the largest orientational errors with heavy upward tails (mean azimuthal error of 49.2 deg and polar angle error of 54.9 deg). The second-lowest orientation error is obtained with Group LASSO (mean azimuthal error of 35.1 deg and polar angle error of 52.8 deg), and the third is the SVD-based HAL1R (mean azimuthal error of 43.7 deg and polar angle error of 48.7 deg). From the basic HAL1R distribution, we see clearly the heavy biasing of the orientation as the azimuthal error has separate concentrations around certain error values and sporadic high bars in polar angle errors. High and uniform angular errors obtained with Fitted-GLASSO demonstrate the importance of embedding SVD in a Bayesian model itself, rather than splitting the localization and orientation estimation tasks, which is possible with fitting methods like UNG and DS. In the radial case, the mean azimuthal angle error is 66.7 degrees and the mean polar angle error is 50.2 degrees.

**Fig. 9:**
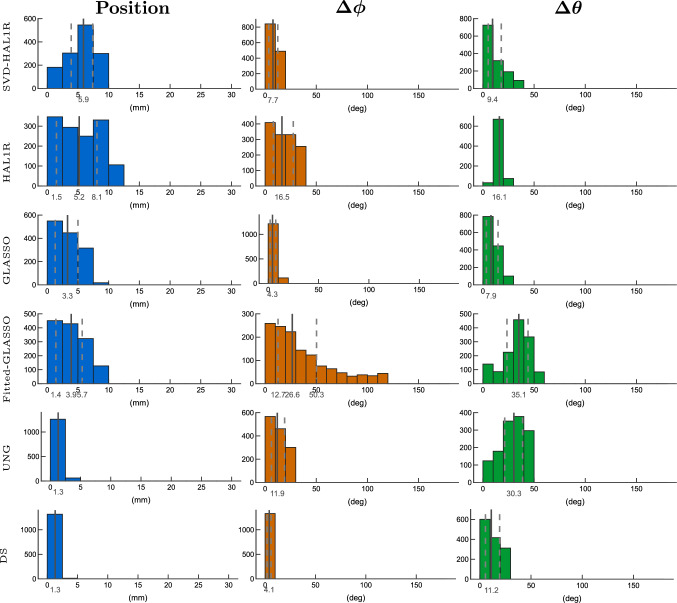
Histogram presentation of dipole estimation errors of 2,000 tangentially oriented cortical sources under 5 % of measurement noise. The mean, first, and third quartiles are presented on the histograms: the mean by a solid line and the other two quartiles by dashed lines. Blue histograms present the dipole localization errors, azimuthal angle errors are displayed by orange histograms, and green histograms provide the polar angle error distribution. Each row is dedicated to an individual source estimation method introduced in Sect. "Inversion Methods". Fitted-GLASSO is the demonstrative method that uses orientationally unbiased Group LASSO to estimate the location after which the orientation is sought out by using SVD

In the tangential case in Fig. 9, the best estimations are obtained with DS. The localization error of UNG and DS are within 1 mm and Group LASSO’s localization error mean is at 3.3 mm. The mean localization error of HAL1R and SVD-based HAL1R are about the same, 5.2 and 5.9 mm in average, but the inter-quartile range of the SVD-based approach is significantly lower, and the distribution lacks the wide tails on both lower and higher ends. The second accurate orientation estimation is obtained with Group LASSO and SVD-based HAL1R, which is the third best by 2 to 3 degrees higher mean azimuthal and polar errors than GLASSO, i.e., 4.1 degrees versus 7.7 degrees and 9.4 degrees versus 7.9 degrees. In both cases, the distributions are one bar (10-degree width) wider for SVD-based HAL1R than GLASSO. Compared to HAL1R, the SVD approach improves the orientation accuracy by about 5 degrees for both orientational errors. Among the main methods, surprisingly, UNG has the worst orientation estimation due to the estimations being too radial, with a mean polar error of 30 degrees. The Fitted-GLASSO has a mean azimuthal angle error of 26.6 degrees and a mean polar angle error of 35.1 degrees, which are significantly higher than those of the compared methods.

**Fig. 10:**
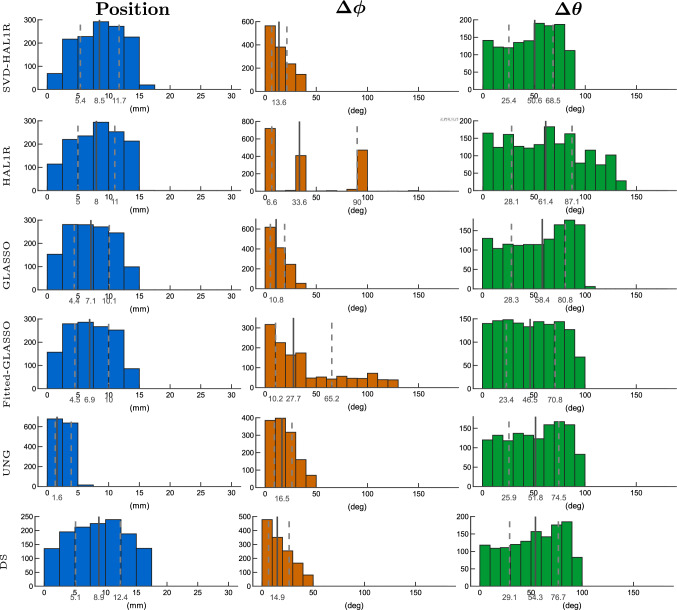
Histogram presentation of dipole estimation errors of 2,000 radially oriented cortical sources under 15 % of measurement noise. The mean, first, and third quartiles are presented on the histograms: the mean by a solid line and the other two quartiles by dashed lines. Blue histograms present the dipole localization errors, azimuthal angle errors are displayed by orange histograms, and green histograms provide the polar angle error distribution. Each row is dedicated to an individual source estimation method introduced in Sect. "Inversion Methods". Fitted-GLASSO is the demonstrative method that uses orientationally unbiased Group LASSO to estimate the location after which the orientation is sought out by using SVD

**Fig. 11:**
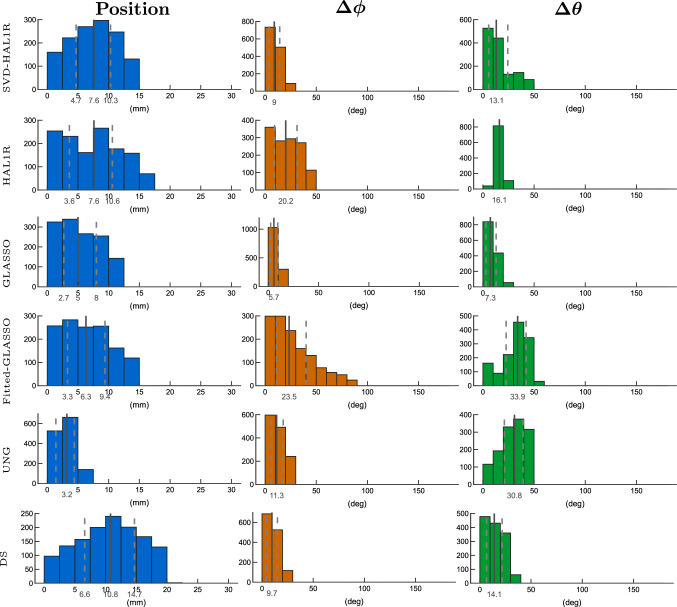
Histogram presentation of dipole estimation errors of 2,000 tangentially oriented cortical sources under 15 % of measurement noise. The mean, first, and third quartiles are presented on the histograms: the mean by a solid line and the other two quartiles by dashed lines. Blue histograms present the dipole localization errors, azimuthal angle errors are displayed by orange histograms, and green histograms provide the polar angle error distribution. Each row is dedicated to an individual source estimation method introduced in Sect. "Inversion Methods". Fitted-GLASSO is the demonstrative method that uses orientationally unbiased Group LASSO to estimate the location after which the orientation is sought out by using SVD

When the noise is set to 15 % in Figs. 10 and 11, a fitting method DS loses its localization accuracy as it is 8.9 mm on average with an interquartile range of 7.3 mm in the radial case, and 10.8 mm on average with an interquartile range of 8.1 mm in the tangential case. UNG, as another fitting method, has the mean error of 1.6 and 3.2 mm in radial and tangential cases, respectively, and therefore, is the most accurate method considering the localization. In the radial case (Fig. 10), the most accurate orientation estimation is obtained with SVD-based HAL1R, which has preserved its orientation estimation accuracy the best (mean azimuthal error of 13.6 deg and polar angle error of 50.6 deg). Group LASSO could be considered as the second best since its azimuthal angle error distribution is narrower than UNG’s azimuthal angle error distribution. GLASSO’s mean azimuthal angle error is 10.8 degrees, while the one with UNG is 16.5 degrees. The polar angle errors are 58.4 degrees with a 43.1 degrees interquartile range and 51.8 degrees with a 48.6 degrees interquartile range, respectively. The polar angle errors for both of them are notably high, as is the case with all of the methods compared. The mean azimuthal angle error of Fitted-GLASSO is 27.7 degrees with an interquartile range of 55 degrees. The method’s polar angle error is 46.5 on average, while the interquartile range is 47.4 degrees.

In tangential cases presented in Fig. 11, the best orientational estimation is obtained with Group LASSO, as its mean azimuthal angle error is 5.7 degrees, the polar angle error is 7.3 degrees, and their interquartile range is less than 10 degrees. SVD-based HAL1R produced the second-most accurate orientation estimates (9 degrees of azimuthal error and 13.1 degrees of polar angle error). In comparison, DS is the close third, with an average azimuthal error of 9.7 degrees and a polar error of 14.1 degrees. The estimations appear significantly more accurate than with the radial sources.

### Real Data

**Fig. 12:**
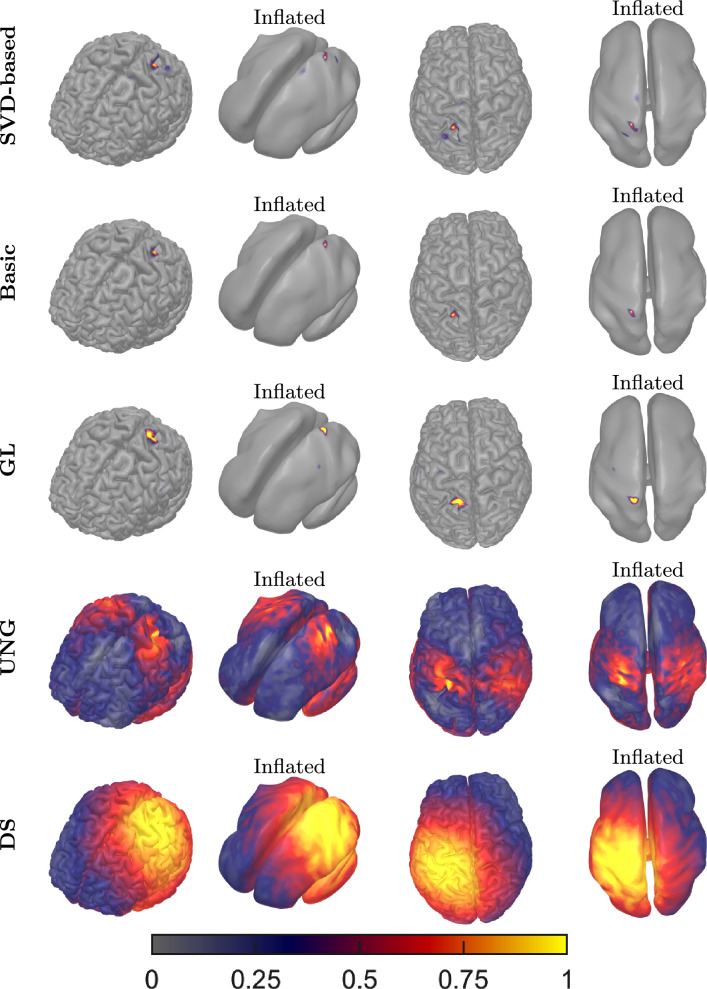
Normalized current density strength fields estimated by the methods compared

With real SEP data, all the methods localized the activity at Brodmann area 3b (Fig. 12). SVD-based HAL1R, Basic HAL1R, GLASSO, and DS estimations are more lateral than UNG. The localized mesh node is exactly the same for all the hierarchical Bayesian methods, and its localized node is the neighbor node of the Dipole Scan’s maximum estimate location. The source extent estimated by the limits the peak on the Brodmann area 3. Similarly, the strongest UNG estimates (red region) cover the Brodmann areas 3 and 4. The estimation peak is reflected in the other hemisphere as well. The SVD-based HAL1R, Basic HAL1R, and GLASSO estimated a strong spike at the sulcus. DS provides a large region of almost equally good locations for the source: the bright yellow region has less than 1 % of the maximum. The DS estimate localizes to the precentral gyrus.

**Fig. 13:**
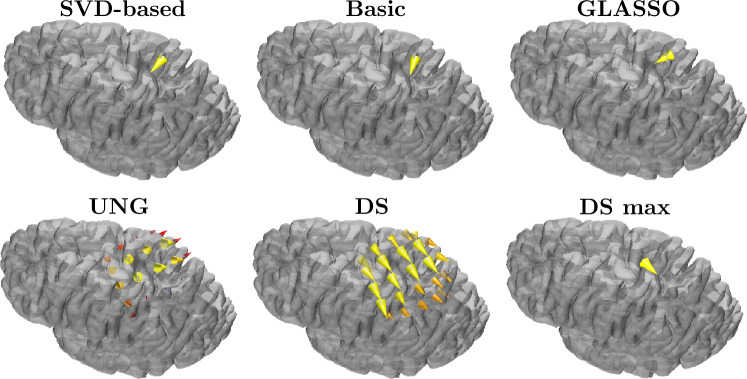
Cone plot representation of the estimated dipolar field given by the different methods at the region of interest around their maximum estimation location. The last image on the bottom right presents the best fit given by DS

In cone plots of the estimated dipole field (Fig. 13), we present the surface dipole field around the maximum magnitude estimation, located at Brodmann 3b for each method. The estimations of the hierarchical Bayesian methods corresponded to one dipole. For DS, we present both the field of highest fitted dipoles and the best fit in separate images, labeled as "DS" and "DS max", respectively. The size and color of each cone represent the relative strength and the apex point in the direction of the estimated dipole at that location.

The estimated dipolar fields are orientationally different for each method. UNG estimates a strongly radial activity, and DS estimates a weakly radial source. SVD-based, Basic HAL1R, and Group LASSO provide congruent anterior-direction estimations. However, it should be noted that basic HAL1R orientation goes along with the Cartesian coordinate of the head model’s coordinate system. The SVD-based HAL1R can be seen to estimate a direction between the orientation estimation of Basic HAL1R and Group LASSO.

## Discussion

In this study, we demonstrate how Singular Value Decomposition (SVD) can be used to improve the source location and, in particular, the source orientation estimation of the heavily orientation-biased Hierarchical Adaptive *L*1-regression (HAL1R). We show how an individual SVD-based 3-coordinate system attached to each source position gives us a set of physiologically meaningful bases that can be used similarly as cortical orientation constraints (Lin et al. 2006) but with a lesser amount of computational effort.

The performance of this enhanced method is then compared to that of orientationally unbiased Adaptive Group LASSO (GLASSO), basic HAL1R, and the Unit noise gain (UNG) beamformer and Dipole scan (DS), two well-established dipole fitting methods for recovering the location and orientation of a dipolar source.

The results show improvement in localization and orientation estimation of SVD-based HAL1R and GLASSO compared to their parent method, HAL1R. The estimations are more stable, i.e., they vary less and are lower on average. While Group LASSO seems to have an advantage in orientational estimation when the source is tangentially oriented, SVD-based HAL1R seems to provide good estimates with narrower error distributions even when the source is radially oriented.

It is studied that dipole fitting methods like UNG and DS are good when the noise level is low, but decrease fast along with the measurement noise (Winkler et al. 2025); similarly, with our experiment, Dipole scan loses its accuracy and UNG loses its orientation estimation accuracy at a higher noise level, unlike the orientation-focused Bayesian methods GLASSO and SVD-based HAL1R as the modeling approach make inferences about the uncertainties regarding the measurement data, forward model, and the brain activity itself. Therefore, the proposed SVD-based HAL1R and Adaptive Group LASSO are considerable alternatives for UNG and DS due to their comparable accuracy and stable performance. Moreover, the advantage of GLASSO and SVD-based HAL1R over UNG and DS is their ability to estimate the source extent, which is accurate based on the estimations made with real P20/N20 potential data. This helps us, e.g., identify the primary somatosensory cortex for clinical purposes (Nitsche et al. 2003; Mohd Zulkifly et al. 2022; Khan et al. 2022).

Assessing the correction of cortical P20/N20 estimates is difficult because of (1) the lack of exact ground truth and (2) the data pre-processing and montage averaging. From the previous studies, we know only that the source should be at Brodmann area 3b, on the posterior wall of the central sulcus, and tangentially located (Hari et al. 1993; Buchner 1995). Assuming that the pyramidal neural cells are normally oriented regarding the grey matter structure (Nitsche et al. 2003), the correct orientation still depends on the exact location in the Brodmann area 3b because of the postcentral gyrus structure. It is also known that the 20 ms somatosensory activity transits within 2 ms to radial P22 activity (Fuchs et al. 1998). Since the measurement data is obtained as a trial average, it is not guaranteed that every spike averaged together is at the same phase. Based on this information, DS is slightly mislocalizing the activity to the posterior wall of the precentral gyrus. The maximum peak of the UNG is on the crown of the postcentral gyrus; however, the estimate has a strong extent to the posterior wall of the central sulcus. The orientation and location of the Group LASSO estimate match exactly the description of the cortical dipolar activity given in the literature, and the SVD-based HAL1R estimate deviates by its direction only slightly while they localize at the same mesh node.

Based on the numerical results, we can expect UNG and DS to be more than 1 cm off when localizing tangential sources, which indicates that the activity should be closer to the longitudinal fissure than the estimated locations given by these methods. Also, if the source has a high radial contribution, as UNG suggests, both UNG and DS should localize the activity almost perfectly and, hence, should have given a congruent estimate, contrary to the obtained results. It is shown by using virtual subjects with different skull and blood conductivity values that filtering-type methods, like UNG and DS here, are sensitive to discrepancies between the used lead field model and the model used to create measurements (Lahtinen et al. 2023). This can also be seen to be true when considering forward modeling and data coming from a real subject. Hence, the distinctness between the model and reality could explain why we cannot fully explain the filtering results obtained with real data using numerical simulations. It was indeed observed with real patient data that fitting methods, such as DS and UNG, are sensitive to modeling errors and discrepancies between the forward model and real measurements (Neugebauer et al. 2022; Rezaei 2021), which could be the reason for mislocalized and incorrectly oriented results.

Based on the statistical results with 2,000 radially and tangentially oriented sources, a significant bias towards tangentially oriented sources is observable for every method except UNG, which is radially biased. The results reveal that the source estimations are not only depth-biased (Fuchs et al. 1999) but also orientation-biased. Even the SVD approach does not mitigate the said bias. A drawback of the SVD approach is its dependence on a forward model that lacks an analytic solution for realistic head models, such as the one used in this study. As the forward model must be obtained by numerical methods, the possible errors in it could cause significant errors in SVD-based orientation estimation.

This article is just one step towards improved orientation estimation by introducing a method to enhance orientation estimation with a focal *L*1-penalized approach, where the focality comes at the cost of highly biased orientation estimations.

As a future study, the orientation and location of the cortical P20/N20 source could be defined more accurately using multiple subjects, realistic multicompartment head models, and SVD-based HAL1R, UNG, and DS for inversion. The approach could be advanced towards unbiased orientation estimation by considering underlying principles in weighting approaches like the one UNG uses or standardization weightings (Pascual-Marqui 2002).

## Conclusions

The SVD-based approach and grouping of 3-dimensional orientational triplets in the prior design can improve the orientation estimation of the HAL1R method, which utilizes a heavily orientation-biased Laplace prior.

## Data Availability

The clinical data used in this study is openly available in http://dx.doi.org/10.5281/zenodo.3888381

## Appendix A: Derivation of Unit Noise Gain Beamformer

The following optimization problem defines the unit noise gain (UNG) beamformer

A1 $$\begin{aligned} \begin{aligned} \min _{{\textbf {w}}_k\in \mathbb {R}^m}&{\textbf {w}}_k^T\Sigma {\textbf {w}}_k\\ \text {subject to }&{\textbf {w}}_k^TL_k\varvec{\theta }=\tau \quad \text {and}\quad {\textbf {w}}_k^T{\textbf {w}}_k=1, \end{aligned} \end{aligned}$$

for some $\tau \in \mathbb {R}$, where $\Sigma =\text {cov}\left[ {\textbf {y}}\right]$ is so-called *error covariance* in the beamformer theory. To simplify the calculations, we consider the solutions $\hat{{\textbf {w}}}$ that belong to the boundary of the 3-dimensional unit ball, i.e, $\hat{{\textbf {w}}}\in \partial \mathbb {B}_3({\textbf {0}};1)$

This type of optimization problem is classically solved by forming the Lagrangian function:

A2 $$\begin{aligned} \mathcal {L}(\hat{{\textbf {w}}},\lambda )=\hat{{\textbf {w}}}^T\Sigma \hat{{\textbf {w}}}+\lambda (\hat{{\textbf {w}}}^TL_k\varvec{\theta }-\tau ) \end{aligned}$$

with the Lagrange multiplier $\lambda>0$. By considering the gradient of the Lagrangian with respect to the $\hat{{\textbf {w}}}$, we obtain

A3 $$\begin{aligned} \nabla _{\hat{{\textbf {w}}}}\mathcal {L}=2\Sigma \hat{{\textbf {w}}}+\lambda L_k\varvec{\theta } \end{aligned}$$

and by seeking out the zero of the gradient, we get

A4 $$\begin{aligned} \hat{{\textbf {w}}}=-\frac{\lambda }{2}\Sigma ^{-1}L_k\varvec{\theta }. \end{aligned}$$

Substituting this into the first constraint,

A5 $$\begin{aligned} -\frac{\lambda }{2}\varvec{\theta }^TL_k^T\Sigma ^{-1}L_k\varvec{\theta }=\tau \end{aligned}$$

giving

A6 $$\begin{aligned} \lambda =\frac{2\tau }{\varvec{\theta }^TL_k^T\Sigma ^{-1}L_k\varvec{\theta }}. \end{aligned}$$

Now, to have $\hat{{\textbf {w}}}\in \partial \mathbb {B}_n({\textbf {0}};1)$, i.e.,

A7 $$\begin{aligned} 1=\hat{{\textbf {w}}}^T\hat{{\textbf {w}}}=\frac{\tau ^2}{\left( \varvec{\theta }^TL_k^T\Sigma ^{-1}L_k\varvec{\theta }\right) ^2}\varvec{\theta }^TL_k^T\Sigma ^{-2}L_k\varvec{\theta }, \end{aligned}$$

must be that

A8 $$\begin{aligned} \tau =\frac{\varvec{\theta }^TL_k^T\Sigma ^{-1}L_k\varvec{\theta }}{\sqrt{\varvec{\theta }^TL_k^T\Sigma ^{-2}L_k\varvec{\theta }}}, \end{aligned}$$

yielding

A9 $$\begin{aligned} \hat{{\textbf {w}}}=\frac{\Sigma ^{-1}L_k\varvec{\theta }}{\sqrt{\varvec{\theta }^TL_k^T\Sigma ^{-2}L_k\varvec{\theta }}}. \end{aligned}$$

That is the UNG filter design.
